# Comparative Study of Preparation, Evaluation, and Pharmacokinetics in Beagle Dogs of Curcumin β-Cyclodextrin Inclusion Complex, Curcumin Solid Dispersion, and Curcumin Phospholipid Complex

**DOI:** 10.3390/molecules27092998

**Published:** 2022-05-07

**Authors:** Wanrong Song, Xizhao Chen, Chongshan Dai, Degui Lin, Xuelin Pang, Di Zhang, Gang Liu, Yipeng Jin, Jiahao Lin

**Affiliations:** 1 College of Veterinary Medicine, China Agricultural University, Beijing 100193, China; wanrong_song@cau.edu.cn (W.S.); daichongshan@cau.edu.cn (C.D.); bs20193050484@cau.edu.cn (D.L.); dzhangdvm@cau.edu.cn (D.Z.); gangliu@cau.edu.cn (G.L.); 2 Beijing Anheal Laboratories Co., Ltd., Beijing 100094, China; chenxizhao@anheal.com (X.C.); pangxuelin@anheal.com (X.P.); 3 Center of Research and Innovation of Chinese Traditional Veterinary Medicine, China Agricultural University, Beijing 100193, China

**Keywords:** curcumin, β-cyclodextrin inclusion complex, solid dispersion, phospholipid complex, bioavailability

## Abstract

Curcumin is a natural acidic polyphenol extracted from turmeric with a wide range of biological and pharmacological effects. However, the application of curcumin for animal production and human life is limited by a low oral bioavailability. In this study, natural curcumin was prepared for the curcumin β-cyclodextrin inclusion complex (CUR-β-CD), curcumin solid dispersion (CUR-PEG-6000), and curcumin phospholipid complex (CUR-HSPC) using co-precipitation, melting, and solvent methods, respectively. Curcumin complex formations were monitored using scanning electron microscopy (SEM), X-ray diffraction (XRD), and Fourier transform infrared (FT-IR) techniques via the shifts in the microscopic structure, molecular structure, and crystalline state. Subsequently, twenty-four female beagle dogs were randomly divided into four groups to receive unmodified curcumin and three other curcumin preparations. The validated UPLC–MS assay was successfully applied to pharmacokinetic and bioavailability studies of curcumin in beagle dog plasma, which were collected after dosing at 0 min (predose), 5 min, 15 min, 30 min, 40 min, 50 min, 1.5 h, 3 h, 4.5 h, 5.5 h, 6 h, 6.5 h, 9 h, and 24 h. The relative bioavailabilities of CUR-β-CD, CUR-PEG-6000, and CUR-HSPC were 231.94%, 272.37%, and 196.42%, respectively. This confirmed that CUR-β-CD, CUR-HSPC, and especially CUR-PEG-6000 could effectively improve the bioavailability of curcumin.

## 1. Introduction

**Figure 1:**
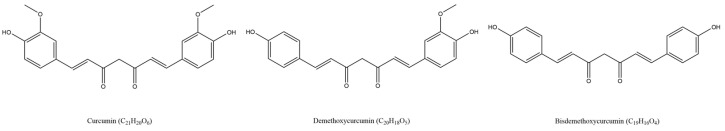
The molecular formula and structural formula of curcumin, desmethoxycurcumin, and bisdemethoxycurcumin.

*Curcuma longa L*, a member of the ginger family (*Zingiberaceae*), which is a rhizomatous herbaceous perennial herb, has been used for thousands of years as a medicine in India, China, and other countries [1]. Curcumin, a yellow low-molecular-weight polyphenolic substance extracted from the rhizomes of *Curcuma longa L*, was first isolated in 1815, but its structure was not elucidated until 1913 [2]. Natural curcumin compounds, which are the main active ingredient in *Curcuma longa L*, mainly consist of curcumin (CUR, 1,7-bis [4-hydroxy-3-methoxy-phenyl]-hepta-1,6-diene-3,5-dione) with its two components, desmethoxycurcumin (CURII, 4-hydroxycinnamoyl- [4-hydroxy-3-methoxycinnamoyl] methane) and bisdemethoxycurcumin (CURIII, bis- [4-hydroxy cinnamoyl] methane) [3,4]. The three ingredients have similar molecular structures (the molecular formula and structural formula of three ingredients are shown in Figure 1), physicochemical properties, and pharmacological effects. To distinguish curcumin compounds from monomers, monomers are denoted by abbreviations below (CUR).

Curcumin has been shown to have a wide range of biological and pharmacological actions. Curcumin exerts its anti-inflammatory effects by inhibiting a variety of pro-inflammatory mediators and chemokines produced by cells in the pathological state, including tumor necrosis factor-α, cyclooxygenase-2, interleukin-18, interleukin-10, and interleukin-6 [5]. In addition, much research has studied the antioxidant effects of curcumin with the mechanism of scavenging activated macrophage superoxide free radicals, hydrogen peroxide, nitric oxide, reduced iron complex, and inhibiting lipid peroxidation [6]. In 1949, Schraufstatter et al. first discovered that curcumin has inhibition properties of various bacteria, including Staphylococcus aureus, Trichophyllum gypsumi, Salmonella paratyphoid, and Mycobacterium tuberculosis [7]. Subsequent studies on the antibacterial activity of curcumin indicated synergism in combination with a majority of antibiotics against Staphylococcus aureus. The antibiotics with a higher synergistic effect with curcumin were amikacin, gentamicin, and ciprofloxacin [8,9]. Recent research has demonstrated that curcumin is active against a plethora of drug-resistant strains and has great potential to be developed as an antibiotic against drug-resistant bacteria in the future [10]. Moreover, curcumin demonstrates other numerous properties such as antitumor, analgesic, antiseptic, chemoprophylaxis, antiviral, and other pharmacological effects [11,12,13,14,15,16,17,18,19].

Various toxicological experiments have confirmed that high doses of curcumin are safe for humans and animals. In a study where 25 volunteers were given up to 8 g of curcumin daily for three months, no obvious signs of poisoning were observed. In five other clinical trials, volunteers took 1125 to 2500 mg of curcumin daily without adverse effects [20]. In animal clinical trials, beagle dogs have been given 4, 2, and 1 g/kg of curcumin multiple times without toxic reactions [21].

It is normally applied as the natural pigment and flavoring agent of food, which results from its extensive pharmacological action and nontoxic characteristics. However, the applications of curcumin in animal production and human life are hampered due to its low water solubility, insufficient oral bioavailability, limited tissue distribution, severe first-pass effect, and short metabolic cycle. Curcumin belongs to the class IV of biopharmaceutics (BCS) that can be easily metabolized by uridine diphosphate glucuronidase transferases in the gastrointestinal tract, leading to a serious reduction in bioavailability. In a study of patients diagnosed with metastatic colorectal cancer, Garcea et al. found that the concentration of curcumin in peripheral circulation only attained a nano-level when patients were given 3.6 g of curcumin daily [22]. Hence, it is crucial to develop new formulations of curcumin to fully exploit its clinical application. Several solutions have been proposed to counter the problem of malabsorption, such as the preparation of emulsions [23], liposomes [24], microcapsules [25], nanoparticles [26], and modification of chemical structures [27]. However, most of them cannot be applied to industrial production, due to difficulties in production, poor quality control, and gastrointestinal toxicity. Among the potential strategies, the cyclodextrin inclusion complex, solid dispersion, and phospholipid complex have emerged as promising strategies to enhance the bioavailability of active constituents, as well as the materials being relatively safe and the preparation being more suitable for industrial production [28,29,30].

The cyclodextrin (CD) is a conical cylinder of an amphiphilic nature, with a hydrophilic outer part and a lipophilic cavity. CD can form host–guest inclusion complexes through weak intermolecular interactions with various guest compounds (organic molecules, inorganic ions, coordination compounds, and even rare gases) [31,32]. Therefore, the stability, solubility, bioavailability, and other physicochemical properties of hydrophobic drugs can be regulated by forming cyclodextrin inclusion complex [33]. At present, the US Food and Drug Administration (FDA) has classified CD as a safe food [34]. Solid dispersion is one of the most successful methods used to improve the dissolution and bioavailability of hydrophobic drugs [29]. Solid dispersion is the water-insoluble compound(s) dispersed in hydrophilic carrier matricin in the form of molecules, colloid, or ultrafine particles, resulting in the release efficiency of the active compound governed by the polymers properties [35]. In 1989, Bombardelli et al. [36] found that polyphenols extracted from plants showed strong affinity with phospholipids in organic solvents, and the polyphenol–phospholipid complex greatly improved the solubility and bioavailability of polyphenols. Amphipathic phospholipids, an important component of biofilm, mainly act as “ushers” of active constituents to help them pass through the outer membrane of gastrointestinal cells, eventually reaching the blood [30,37]. After forming phospholipid complexes, the membrane permeability and oil–water partition coefficient of constituents are significantly increased. In addition, several studies have proved the beneficial roles of three preparations in enhancing the bioavailability of insoluble drugs [28,30,31,35,38].

In this study, high-performance liquid chromatography (HPLC) and ultra-performance liquid chromatography–mass spectrometry (UPLC–MS) were selected as detection methods of curcumin in vitro and in vivo. Subsequently, we established and optimized the preparation of the curcumin β-cyclodextrin inclusion complex, curcumin solid dispersion, and curcumin phospholipid complex. Finally, the optimum preparation that enhanced the bioavailability of curcumin was screened out by a pharmacokinetic study in beagle dogs.

## 2. Results

### 2.1. Establishment of HPLC Methodology

The HPLC chromatograms of blank matrix, mixing standard of CUR, CUR II, and CUR III, and a random sample indicated no interference in the simultaneous detection of CUR, CUR II, and CUR III. The linearity of the calibration curve was evident over the concentration of mixed standard solutions of CUR, CUR II, and CUR III ranges of 10–200 μg/mL with r^2^ > 0.995 in all validation runs. The representative calibration curve of CUR III was Y = 187478X − 427707 (r^2^ = 0.9996), that of CUR II was Y = 190952X − 405703 (r^2^ = 0.9996), and that of CUR was Y = 167519X − 259650 (r^2^ = 0.9998). X and Y represent the nominal concentration and the peak area of CUR, CUR II, and CUR III, respectively. The results of precision, repetition, and average recoveries showed that the relative standard deviations (RSDs) of the nominal concentration of CUR, CUR II, and CUR III were all less than 2.0%, and the average recoveries at high, medium, and low concentrations were in the range of 95–105%. In conclusion, the HPLC method can accurately and reliably measure CUR, CUR II, and CUR III simultaneously.

### 2.2. Optimization of Three Curcumin Preparations

**Figure 2:**
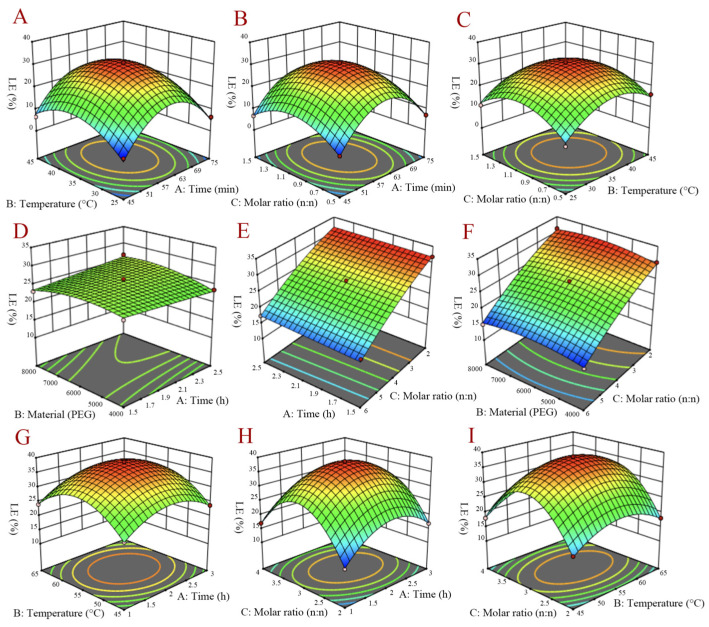
3D-response surface of interaction between random two-factor on curcumin β-cyclodextrin inclusion complex (**A**–**C**), curcumin solid dispersion (**D**–**F**), and curcumin phospholipid complex (**G**–**I**).

Based on the results of the one-factor experiment, a total number of 15 runs of experimental conditions including three center points were selected for different combinations for statistical analysis of BBD. The suitability of the model to predict the optimum response value for the preparation technology was evaluated using the optimal conditions chosen. Variance analysis of the three models showed that there was no statistically significant difference in the Lack of Fit, suggesting that the model was fit. The three-dimensional curves of three curcumin preparations are demonstrated in Figure 2. Combined with the clinical demands and production costs, preparation parameters of three curcumin preparations were optimized as follows: (1) CUR-β-CD: an inclusion time of 60 min, an inclusion temperature of 36 °C, and a 1:1 molar ratio of β-CD to CUR; (2) CUR-PEG-6000: a mixing time of 2 h, carrier material using PEG-6000, and a 4:1 molar ratio of PEG-6000 to curcumin; (3) CUR-HSPC: an inclusion time of 2 h, an inclusion temperature of 56 °C, and a 3:1 molar ratio of HSPC to CUR.

### 2.3. Characterization of Three Preparations of Curcumin

The three optimized preparations of curcumin complexes were characterized by SEM, FT-IR, and X-ray diffraction.

#### 2.3.1. Results of Scanning Electron Microscopy

**Figure 3:**
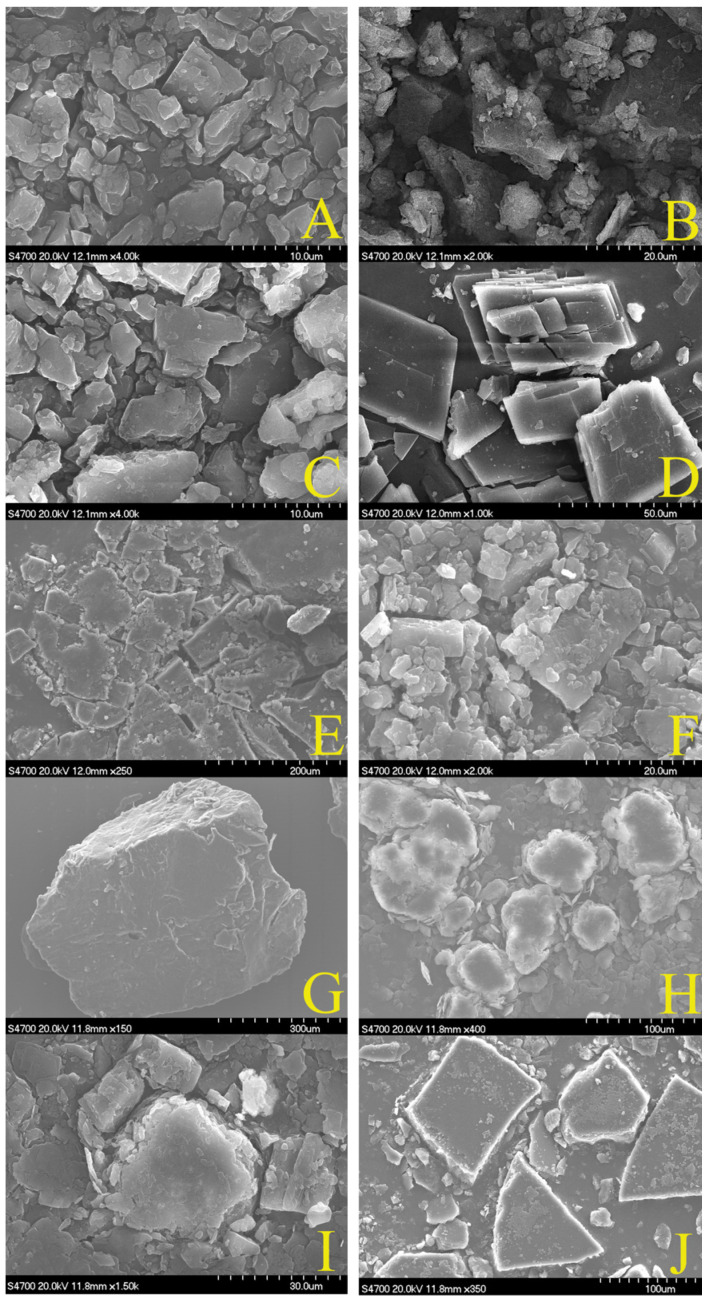
The SEM images of curcumin β-cyclodextrin inclusion complex, curcumin solid dispersion, and curcumin phospholipid complex: (**A**) curcumin, (**B**) β-CD, (**C**) physical mixture of β-CD and curcumin, (**D**) CUR-β-CD, (**E**) PEG-6000, (**F**) physical mixture of PEG-6000 and curcumin, (**G**) CUR-PEG-6000, (**H**) HSPC, (**I**) physical mixture of HSPC and curcumin, (**J**) CUR-HSPC.

Figure 3 indicates that the surface micro-structure of the three curcumin preparations all changed observably. The SEM images of the physical mixture of curcumin and β-CD, PEG-6000, and HPSC, respectively, demonstrated that the morphology was only the mixed structure of curcumin and the carrier, and the original morphology of both had not changed. However, in the SEM image of curcumin β-cyclodextrin inclusion complex, the original morphology of the curcumin and β-cyclodextrin disappeared and showed a uniform plate structure. Compared with the raw material composition, the curcumin solid dispersions were irregularly massive with a larger particle size. The SEM images of the curcumin phospholipid complex showed that the micro-structure of the two compounds had changed to a triangular or square block structure with a larger particle size. The SEM images of the three preparations all showed the formation of a new morphology.

#### 2.3.2. Results of Fourier Transform Infrared

**Figure 4:**
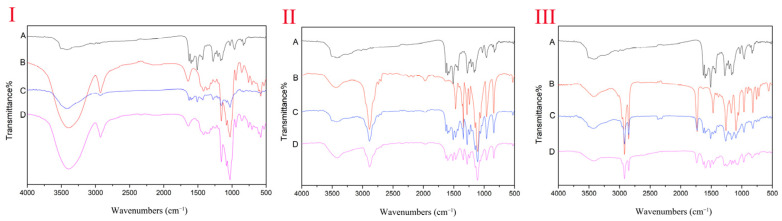
The FT-IR spectrum of curcumin β-cyclodextrin inclusion complex, curcumin solid dispersion, and curcumin phospholipid complex: (**I**-A) curcumin, (**I**-B) β-CD, (**I**-C) physical mixture of β-CD and curcumin, (**I**-D) CUR-β-CD, (**II**-A) curcumin, (**II**-B) PEG-6000, (**II**-C) physical mixture of PEG-6000 and curcumin, (**II**-D) CUR-PEG-6000, (**III**-A) curcumin, (**III**-B) HSPC, (**III**-C) physical mixture of HSPC and curcumin, (**III**-D) CUR-HSPC.

The FT-IR spectrum of curcumin (Figure 4) showed the significant peaks at 3509 cm^−1^, 3420 cm^−1^ (phenolic -OH stretching vibration); 1628 cm^−1^ (enol-carbonyl stretching vibration); 1600 cm^−1^ (benzene ring stretching vibration); 1509 cm^−1^ (C=O vibration); 1429 cm^−1^ (olefin C-H bending vibration); 1276 cm^−1^, 1025 cm^−1^ (C-O stretching bands). The FT-IR spectrum of the physical mixture of curcumin and the carrier in the three preparations was semblable, showing a simple superposition of two components without the interaction of chemical bonds, while the FT-IR spectrum of CUR-β-CD was similar to that of β-CD (Figure 4) owing to the interaction between curcumin and the CD. However, β-CD almost covered the characteristic peak of curcumin in the range of 800 cm^−1^ to 4000 cm^−1^, which led to the disappearance of the characteristic peak of curcumin. At the same time, there was no new absorption peak compared with β-CD, indicating that the inclusion process was a physical process rather than a chemical process, which belongs to a nonbonded complex. The FT-IR spectrum of the curcumin solid dispersion group is illustrated in Figure 4. We found that the characteristic absorption peaks of the 3509 cm^−1^ in curcumin disappeared and 1342 cm^−1^ and 1282 cm^−1^ in PEG-6000 were slightly offset, indicating that -OH in curcumin might be associated with -CH_2_ in PEG-6000 through hydrogen bonding. In the FT-IR spectrum of the curcumin phospholipid complex group (Figure 4), the characteristic absorption peak of HSPC shifted from 1259 cm^−1^ to 1283 cm^−1^, and the 3509 cm^−1^ in curcumin disappeared. It was confirmed that the -OH in curcumin concatenated to P=O in HSPC by the intermolecular force or hydrogen bond.

#### 2.3.3. Results of X-Ray Diffraction

**Figure 5:**
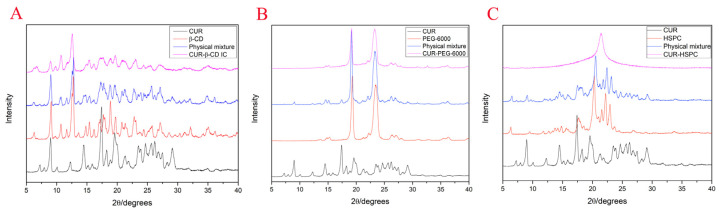
The XRD curves of curcumin β-cyclodextrin inclusion complex (**A**), curcumin solid dispersion (**B**), and curcumin phospholipid complex (**C**).

The X-ray diffractogram of CUR-β-CD, CUR-PEG-6000, and CUR-HSPC (Figure 5) showed similar trends of crystal state changes compared with unmodified curcumin. In each group, the X-ray diffractogram of the simple mixture of curcumin and the carrier was the sum of the spectral lines of both components that were present, as expected.

However, in the curcumin β-cyclodextrin inclusion complex, curcumin solid dispersion, and curcumin phospholipid complex, the diffraction peaks of curcumin changed from sharp to smooth or even disappeared, indicating that the crystal state of curcumin gradually changed from a crystal to amorphous state.

### 2.4. Establishment of UPLC–MS Methodology

**Figure 6:**
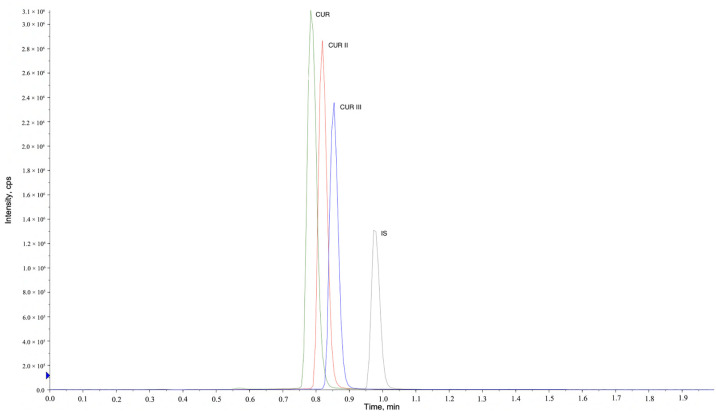
Representative multiple-reaction monitoring chromatograms of CUR, CUR II, CUR III, and IS.

The selectivity of the assay was verified by comparing the MRM chromatograms of the drug-containing samples and the blank samples. No interferences from the blank beagle dog plasma samples were observed at the corresponding times for CUR, CUR II, CUR III, and IS (Figure 6). The retention times of CUR, CUR II, CUR III, and IS were 0.73, 0.77, 0.80, and 0.99 min, respectively.

Analytes at seven nonzero calibration points, ranging from 0.125 to 20 ng/mL, were used to prepare the calibration curve and evaluate the linearity. A weighted (1/x^2^) least squares regression analysis was performed by plotting the peak area ratio (analyte/IS) against the nominal plasma concentration. The result indicated that the correlation coefficient (r) of the calibration curves of CUR, CUR II, and CUR III was all greater than 0.995. The inter- and intra-day precision and accuracy of CUR, CUR II, and CUR III for LLOQ and QC samples at three concentration levels indicated that the deviations in four batches were less than 15%. The mean extraction recovery was in the range of 94.61–100.66% at the concentration levels of 0.3, 5, and 15 ng/mL, suggesting that the extraction efficiency of the method is acceptable with acetonitrile as a protein precipitant. The bias of the stability samples ranged from −1.03% to 3.67% when the QC samples were stored at 4 °C for 12 h. The matrix effect ranged from 95.40% to 105.10% in beagle dog plasma, indicating the absence of a matrix effect. The residue results showed that the peak areas of CUR, CUR II, and CUR III were all less than 20% of the LLOQ. To sum up, the UPLC–MS method can detect the concentration of CUR, CUR II, and CUR III in beagle dog plasma accurately and efficiently.

### 2.5. Pharmacokinetic Study

**Figure 7:**
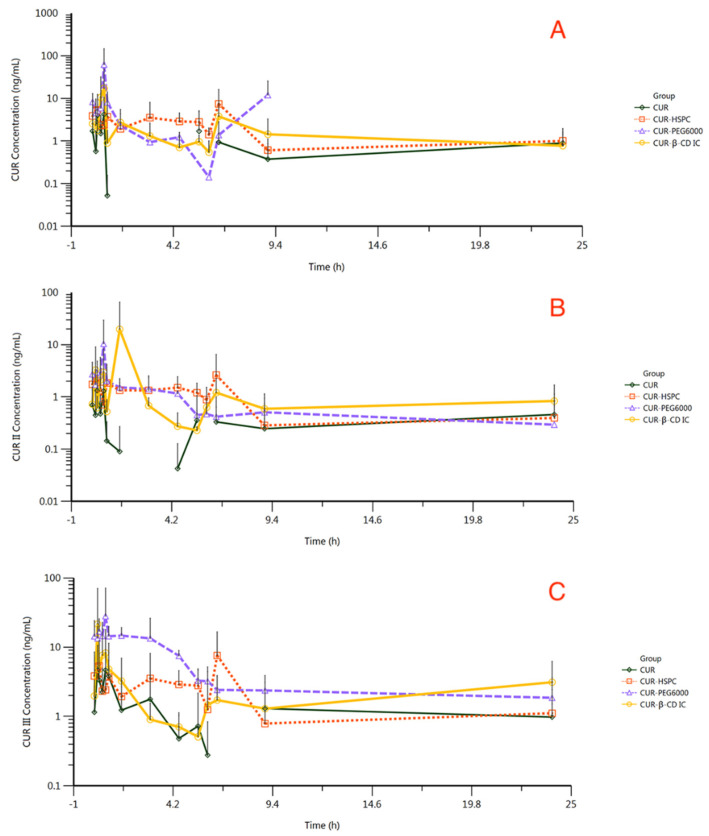
The average concentration of CUR (**A**), CUR II (**B**), and CUR III (**C**) in beagle dogs’ plasma of all groups within 24 h after administration (values reported as mean ± S.D., logarithmic representation, n = 6).

The validated UPLC–MS method was applied to a pharmacokinetic study of CUR, CUR II, and CUR III in beagle dog plasma. The average plasma concentrations of CUR, CUR II, and CUR III in beagle dog vs. time profiles are depicted in Figure 7 and the main calculated pharmacokinetic parameters are summarized in Table 1 through noncompartment (NCA) model analysis. After oral administration (150 mg/kg), CUR was absorbed into the blood from the gut, and it reached the maximum plasma concentration (4.22 ng/mL) at 40 min post-dose, which rapidly dropped to extremely low levels after. The mean concentrations of CUR II and CUR III peaked at 1.32 ng/mL and 4.68 ng/mL at 20 min and 15 min, respectively, similar to CUR. Compared with unmodified curcumin, the peak concentrations of CUR, CUR II, and CUR III in the CUR-β-CD group, CUR-PEG-6000 group, and CUR-HSPC group were significantly increased. For instance, the peak concentration of CUR in the CUR-PEG-6000 group was as high as 62.09 ng/mL, indicating that the three preparations could increase the blood concentration of curcumin. In this experiment, WinNonlin was used for NCA data analysis. The t_1/2_ was obtained by calculating the “terminal elimination half-life”. It is based on the principle of statistical moments to process the data (it calculates the t_1/2_ after calculating the slope at the end time point by default). Due to the extremely poor absorption of CUR and CUR II in the CUR group, the concentration at some time points was lower than the LLOQ, and the drug concentration–time curve was not recorded, so the t_1/2_ could not be calculated. Therefore, the t_1/2_ data of CUR and CUR II in related studies were compared with the t_1/2_ data of three preparations in this study. According to studies on the pharmacokinetics of curcumin, the oral t_1/2_ of CUR in beagle dogs is about 0.85 h [39]. Because of the lack of relevant pharmacokinetic studies on CUR II in beagle dogs, the pharmacokinetic studies on oral CUR II in SD rats was selected as a reference. Correlational research revealed that the t_1/2_ of oral CUR II was about 5.5 h [40]. In addition, the T_max_ of CUR, CUR II, and CUR III in CUR-β-CD, CUR-PEG-6000, and CUR-HSPC was longer than that of unmodified curcumin. Thus, the drug of CUR-β-CD, CUR-PEG-6000, and CUR-HSPC will be circulated for a longer time. The relative bioavailability of curcumin in CUR-β-CD, CUR-PEG-6000, and CUR-HSPC was calculated with unmodified curcumin as the reference preparation. The relative bioavailability of CUR-β-CD, CUR-PEG-6000, and CUR-HSPC was 231.94%, 272.37%, and 196.42%, respectively. Thus, there were increases in the bioavailability, which was accompanied by a longer t_1/2_ in the CUR-β-CD, CUR-PEG-6000, and CUR-HSPC vs. unmodified curcumin.

## 3. Discussion

In this study, curcumin was prepared for the curcumin β-cyclodextrin inclusion complex, curcumin solid dispersion, and curcumin phospholipid complex using co-precipitation, melting, and solvent methods, respectively. The physicochemical property of curcumin complex formations was monitored using SEM, XRD, and FT-IR. FTIR is a sensitive methodology and most chemical changes can be detected by this method [41]. FT-IR spectra showed modifications of the peaks of phenolic -OH of curcumin, which agree with the studies of Patro et al. [42] and Wan et al. [43]. Subsequently, another important characterization of the curcumin in three preparations is the crystallinity based on XRD. The main peaks of curcumin are consistent with the diffraction pattern of curcumin proposed by Patro et al. [42] and Paradkar et al. [44].

Currently, the pharmacokinetics of orally administered curcumin have been characterized in rats and humans. However, the pharmacokinetics of the orally administered β-cyclodextrin inclusion complex, curcumin solid dispersion, and curcumin phospholipid complex have rarely been expounded in beagle dogs. In this study, curcumin and the three preparations show a multi-peak phenomenon in the drug concentration–time curve. The multi-peak in the blood fluid concentration–time curve is usually due to gavage or oral administration, which can occur because of various mechanisms. These factors are related to the physiological makeup of the gastrointestinal tract. This includes absorption from multiple parts of the gastrointestinal tract, biochemical differences in the different regions of the gastrointestinal tract, and the pH and components of bile [45]. One of the most common sources of multi-peaks is “enterohepatic recycling”. Biliary excreted drugs or other substances enter the small intestine, where they are absorbed by intestinal cells and transported back to the circulation of the liver. The multi-peak phenomenon will occur if the number of reabsorbed drugs is extensive, leading to an increased drug concentration in the blood. Studies have shown that oral curcumin is subjected to a certain degree of enterohepatic recycling [46]. Another contributing factor is related to the formulation, such as excipients incorporated into the preparations. If free curcumin is adsorbed onto the surface of the preparations, it may lead to an “explosive effect” and form the first peak of drug release. In addition, if there are immediate-release or sustained-release ingredients from the preparation, multimodality may also occur because of the differences in the release rates.

Greater amounts of CUR, CUR II, and CUR III were observed in the absorptive phase following administration of the three preparations as compared to unmodified curcumin. Due to the pharmacokinetics of orally administrated curcumin having rarely been characterized in dogs, we compared the studies with the pharmacokinetics of orally administrated curcumin in rats. In a study of rats, plasma levels were only 2.4 ng/mL after 30 min of administration of curcumin at 340 mg/kg [47]. Teixeira et al. [41] orally administered 500 mg/kg of curcumin to rats and, at 30 min, they reached a maximal plasma concentration of 3.2 ng/mL. In contrast, the curcumin solid dispersion, of which the maximum plasma concentration reached 17.6 ng/mL at 60 min after oral gavage, was approximately fivefold higher than that of unformulated curcumin. In our study, after oral administration of unformulated curcumin at 150 mg/kg for beagle dogs, CUR, CURII, and CURIII reached the maximum plasma concentration of 4.22 ng/mL, 1.32 ng/mL and 4.68 ng/mL at 40 min, 20 min, and 15 min post-dose, respectively, while the maximum plasma concentration of CUR, CURII, and CURIII in the curcumin solid dispersion peaked at 62.09 ng/mL, 10.37 ng/mL, and 28.05 ng/mL, which were approximately 15-fold, 8-fold, and 6-fold higher than that of unmodified curcumin, respectively. The curcumin phospholipid complex yielded a relative bioavailability of 196.42% as compared to unformulated curcumin, which was similar to the 206.56% increase noted previously by Wang et al. [48]. The speculated reasons of bioavailability increasing in curcumin are as follows: (1) The above “Section 2.3.2” indicates that curcumin has been embedded in the β-CD cavity to form a stable nonbonding complex. Curcumin stability improved significantly owing to the barrier effect of β-CD in the inclusion complex. Moreover, as a lipophilic drug, curcumin is mainly transported passively when absorbed across membranes. Studies have shown that the penetration rate of lipophilic drugs in the mucous layer of the mucosa increases after β-CD inclusion to increase the solubility of the drug outside the cell membrane [49,50]. Additionally, β-CD can reduce interstitial cholesterol and affect the tight junction protein between cells [51]. (2) Amorphous curcumin was uniformly dispersed in PEG-6000 after the curcumin solid dispersion was formed, which ensured the high water dispersibility of curcumin. As an excellent water-soluble carrier, PEG-6000 improved the wettability of weakly hydrophilic curcumin. As PEG-6000 dissolved quickly, the highly dispersed curcumin was wetted, increasing its dissolution rate and solubility in the gastrointestinal environment [52,53]. (3) A more stable complex with a specific structure of drug molecules was formed through charge migration under certain conditions because the oxygen atom in the phospholipid molecule P=O has a strong tendency to gain electrons. Amphipathic phospholipids mainly act as “ushers” of active constituents to help poorly soluble drugs pass through the outer membrane of gastrointestinal cells, improving the membrane permeability and oil–water partition coefficient of drugs [37,54,55]. In addition, the increase in the plasma drug concentration is usually caused by an increase in the solubility and dissolution rate of the drug in the gastrointestinal fluid. These are consistent with the relevant research on the improvement of the solubility and bioavailability of poorly soluble drugs through the three preparations.

## 4. Materials and Methods

### 4.1. Materials

Curcumin (a mixture of CUR, CUR II, and CUR III, total curcuminoid content > 95%) was provided by Baoji Guokang Bio-Technology Co., Ltd., (Baoji, China). Standard CUR, standard CUR II, standard CUR III, and standard emodin were purchased from the National Institutes for Food and Drug Control (Beijing, China). Hydrogenated soy phosphatidylcholine was produced by AVT Pharmaceutical Tech Co., Ltd., (Shanghai, China). β-cyclodextrin, polyethylene glycol (average Mn 4000, 6000, 8000, and 10,000), methanol, and acetonitrile (Chromatographic grade) were obtained from Shanghai Macklin Biochemical Co., Ltd., (Shanghai, China). Twenty-four female beagle dogs (10~15 kg) were supplied by Beijing Xinuogu Biological Technology Co., Ltd., (Beijing, China).

### 4.2. Determination of Curcumin Content In Vitro

#### HPLC Conditions

The HPLC method was established on a Shimazu LC-20A high-performance liquid chromatograph with an SPD-20A UV detector. Chromatographic separation was executed on an Eclipse XDB-C18 column (150 mm × 4.6 mm, 5 μm; Agilent Technologies Co., Ltd., Beijing, China) kept at a temperature of 30 °C, with water containing 0.1% phosphoric acid (A) and acetonitrile (B) as a mobile phase. The elution was carried out with a flow rate of 0.6 mL/min, and a wavelength of 430 nm was used for detection. The injection volume was 10 μL. The related parameters including selectivity, linearity, accuracy and precision, and recovery were completely assessed.

### 4.3. Preparation of Three Curcumin Preparations

#### 4.3.1. Curcumin β-Cyclodextrin Complex

The curcumin β-cyclodextrin inclusion complex was prepared by the saturated aqueous solution method. Briefly, different proportions of β-cyclodextrin (β-CD) and curcumin were dissolved in deionized water and absolute ethanol, respectively (one gram of β-CD is dissolved in 25 mL of deionized water; one gram of curcumin is dissolved in 115 mL of anhydrous ethanol). The curcumin solution was slowly dropped into β-CD solution and mixed using a SN-MS-H280D magnetic stirrer (Shanghai Shangyi Instrument Equipment Co., Ltd., Shanghai, China) at a certain rotational speed at a certain temperature. After stirring, it was placed in the refrigerator at 4 °C for 24 h for precipitation and crystallization. A SHB-IIIA vacuum pump (Shanghai Zhenjie Experimental Equipment Co., Ltd., Shanghai, China) was used for filtration and then dried after washing with anhydrous ethanol.

#### 4.3.2. Curcumin Solid Dispersion

The melting method was selected for preparing the curcumin solid dispersion. Different proportions of curcumin and the carrier (polyethylene glycol (PEG) 4000, PEG-6000, PEG-8000, and PEG-10000) were mixed at 65 °C with a SN-MS-H280D magnetic stirrer at a certain rotational speed. This stirred melt was rapidly poured onto the glass plate to cool into a thin layer of solid, which was put in the refrigerator at −20 °C. After 24 h, the solid was ground to obtain the curcumin solid dispersion.

#### 4.3.3. Curcumin Phospholipid Complex

The curcumin phospholipid complex was obtained by the solvent method. Hydrogenated soy phosphatidylcholine (HSPC) and curcumin with different molar ratios were dissolved in anhydrous ethanol and stirred at a certain temperature with a SN-MS-H280D magnetic agitator. The resulting solution was taken in a 500 mL round-bottom flask, and a RE-2000A rotary evaporator (Shanghai Yarong Biochemical Instrument Factory, Shanghai, China) was used to remove the anhydrous ethanol. The residual material was dissolved in n-hexane. The curcumin phospholipid complex was precipitated by centrifuged at 3000 r for 5 min. The supernatant was collected and rotated to remove n-hexane and dried to remove traces of solvents.

### 4.4. Optimization of Three Curcumin Preparations

The influences of inclusion temperature, inclusion time, molar ratio, and rotational speed on the optimization scheme of the curcumin β-cyclodextrin inclusion complex and curcumin phospholipid complex were studied by a one-factor experimental design with the loading efficiency (*LE*) as the index. For the curcumin solid dispersion, different carriers, inclusion times, molar ratios of the carrier and curcumin, and rotational speeds were selected as the evaluation indicators. Whereafter, according to the results of the one-factor experiment, influential factors were selected for the Box–Behnken design (BBD) to attain the optimal preparation technology, which used Design Expert Software V 11.0 to analyze and collect data from the experimental runs.

*LE* (*%*) = *We*/*Wm* × *100%* (1)

*We* represents the weight of the drugs loaded, while *Wm* represents the total weight of the preparation.

### 4.5. Characterization of Three Curcumin Preparations

Each preparation was divided into four groups: curcumin group, carrier group, physical mixture of curcumin and carrier group, and curcumin preparation group. The surface morphology and microscopic structure characterization of each sample were observed by an S4700 scanning electron microscope (SEM, Hitachi, (China) Ltd., Beijing, China). Samples were prepared by fixed dry particles onto the copper plate base with conductive tape followed by injecting for test after spraying gold in an argon environment. The operating voltage was 20 kV. The images were magnified to different multiples for observation.

The molecular structure of all samples was monitored with a Nicolet 6700 Fourier transform infrared (FT-IR) spectrometer (Thermo Fisher Scientific (China) Co., Ltd., Shanghai). The grounded samples were mixed with KBr powder and pressed into discs. The spectral range was 400–4000 cm^−1^ with 32 scans and a resolution of 1 cm^−1^.

Moreover, an Ultima IV X-ray diffractometer (XRD, Rigaku Beijing Corporation, Beijing, China) was used to analyze the crystalline state of all samples. The samples were investigated in the 2*θ* range of 5°–40° with the Cu target. The tube pressure was 40 kV, the tube flow was 100 mA, the divergence slit was 1°, the scattering slit was 1°, the receiving slit was 0.3 mm, and the scanning amplitude was 0.02°.

### 4.6. Pharmacokinetics of Beagle Dogs In Vivo

#### 4.6.1. UPLC–MS Conditions

The UPLC–MS method was used to determine the curcumin concentration in biological matrices. It was established on a Waters ACQUITY UPLC I-Class system (Waters Technologies (Shanghai) Ltd., Shanghai, China) coupled to an AB SCIEX 5500 triple quadrupole mass spectrometer (AB Sciex Pte. Ltd., Shanghai, China). Chromatographic separation was executed on a Shim-pack Scepter C18-120 column (2.1 mm × 50 mm, 1.9 μm; Shimadzu (China) Co., Ltd., Shanghai, China) kept at a temperature of 50 °C, with water (95% water + 5% acetonitrile) containing 5 mmoL of ammonium acetate (A) and acetonitrile containing 0.1% formic acid (B) as a mobile phase. The gradient elution was optimized as follows: 40–100% B at 0–1.0 min; 100–40% B at 1.0–1.1 min; 40% B at 1.1–2.0 min. The flow rate was 0.4 mL/min. The injection volume was 2 μL.

The UPLC system was connected to the mass spectrometer via an electrospray ionization (ESI) interface operating in negative ion mode. Emodin was used as an internal standard (IS). The capillary temperatures were set at 500 °C. The declustering potential was maintained at −60 V. The collision energy was set at −15 V for both CUR and CUR II, −33 v for CUR III, and −35 V for IS. The multiple reaction monitoring (MRM) mode was employed for the determination with precursor-to-product ion transitions at *m/z* 367.1 > 217.0, *m/z* 337.1 > 217.0, and *m/z* 307.1 > 119.0 for CUR, CUR II, and CUR III, respectively. The IS was set at *m/z* 269.0 > 225.0. The related parameters including selectivity, carry-over, linearity, accuracy and precision, recovery, matrix effect, and stability under different conditions were completely assessed.

#### 4.6.2. Detection of Beagle Dog Plasma Samples

##### Group and Administration of Beagle Dogs

Twenty-four female beagle dogs weighing 10~15 kg for the full set of pharmacokinetics were used in this study. All animal procedures were carried out after obtaining approval from the “China Agricultural University Laboratory Animal Welfare and Animal Experimental Ethical Committee (Approval ID: AW30111202-1-1)”. The total number of beagle dogs were randomly divided into four groups to receive the unmodified curcumin, curcumin β-cyclodextrin inclusion complex, curcumin solid dispersion, or curcumin phospholipid complex at a dose of 150 mg/kg orally. All beagle dogs were fasted 12 h before oral drug capsules. The 0.5 mL blood samples were collected into EDTA tubes after dosing at 0 min (predose), 5 min, 15 min, 30 min, 40 min, 50 min, 1.5 h, 3 h, 4.5 h, 5.5 h, 6 h, 6.5 h, 9 h, and 24 h, which were centrifuged at 3500 r/min for 10 min in a 4 °C microcentrifuge, and the supernatant of each was collected and kept at −80 °C until analysis.

##### Pretreatment of Beagle Dog Plasma Samples

An amount of 50 μL of beagle dog blank plasma was precisely absorbed, and 200 μL of acetonitrile (including internal standard) was added after vortex mixing. Then, the supernatant was taken and injected into the UPLC–MS system after being centrifuged at 12,000 r/min for 10 min. All the data acquisition was obtained by Analyst 1.6.2 AB SCIEX software. The pharmacokinetic parameters were calculated using Phoenix WinNonlin 8.2.0.

## 5. Conclusions

In this study, we obtained the optimal preparation technology of the curcumin β-cyclodextrin inclusion complex, curcumin solid dispersion, and curcumin phospholipid complex, which are as follows: (1) CUR-β-CD: an inclusion time of 60 min, an inclusion temperature of 36 °C, and a 1:1 molar ratio of β-CD to CUR; (2) CUR-PEG-6000: a mixing time of 2 h, carrier material using PEG-6000, and a 4:1 molar ratio of PEG-6000 to curcumin; (3) CUR-HSPC: an inclusion time of 2 h, an inclusion temperature of 56 °C, and a 3:1 molar ratio of HSPC to CUR. Subsequently, a simple and sensitive UPLC–MS assay was developed and validated for the determination of CUR, CUR II, and CUR III in beagle dogs. The pharmacokinetic results confirmed that CUR-β-CD, CUR-HSPC, and especially CUR-PEG-6000 could effectively improve the bioavailability of curcumin.

## Figures and Tables

**Table 1:** The main pharmacokinetic parameters of CUR, CUR II, and CUR III in beagle dogs’ plasma of all groups (values reported as mean ± S.D., n = 6).

Components	Parameters	Unmodified Curcumin	CUR-β-CD	CUR-PEG-6000	CUR-HSPC
CUR	t_1/2_(h)	/	22.45 ± 30.48	1.60 ± 0.92	8.25 ± 5.50
	C_max_(ng/mL)	7.95 ± 3.71	25.49 ± 27.97	35.03 ± 61.29	12.66 ± 6.16
	T_max_(h)	0.35 ± 0.24	2.72 ± 2.95	1.96 ± 3.46	2.61 ± 2.86
	AUC_0-24h_(h·ng/mL)	14.42 ± 15.19	31.26 ± 20.98	47.07 ± 79.84	38.31 ± 14.66
	V_d_(L/kg)	/	88,116.10 ± 80,285.16	167,082.26 ± 266,551.49	40,667.88 ± 12,737.62
	CL(L/h/kg)	/	6103.77 ± 4234.69	48,352.49 ± 67,003.59	4554.86 ± 3103.81
CUR II	t_1/2_(h)	/	27.64 ± 32.54	7.12 ± 5.78	9.05 ± 5.80
	C_max_(ng/mL)	2.37 ± 0.88	22.71 ± 44.48	11.44 ± 18.46	5.42 ± 3.01
	T_max_(h)	0.35 ± 0.24	2.89 ± 2.82	0.39 ± 0.23	1.38 ± 2.52
	AUC_0-24h_(h·ng/mL)	7.19 ± 9.61	46.36 ± 86.65	10.45 ± 8.11	18.31 ± 11.27
	V_d_(L/kg)	/	309,845.96 ± 219,733.66	89,392.67 ± 75,834.45	100,434.21 ± 56,030.46
	CL(L/h/kg)	/	14,751.09 ± 11,858.04	9268.27 ± 2708.14	9868.20 ± 6601.76
CUR III	t_1/2_(h)	5.69 ± 5.27	7.50 ± 3.82	11.76 ± 14.20	7.49 ± 6.34
	C_max_(ng/mL)	7.24 ± 5.71	27.03 ± 46.28	38.15 ± 38.13	12.84 ± 6.47
	T_max_(h)	0.31 ± 0.25	5.35 ± 9.40	1.54 ± 1.20	2.61 ± 2.86
	AUC_0-24h_(h·ng/mL)	27.83 ± 39.03	37.05 ± 37.68	77.14 ± 43.37	40.49 ± 19.88
	V_d_(L/kg)	62,476.37 ± 795.00	69,737.54 ± 46,305.10	13,509.02 ± 8797.05	35,460.51 ± 19,344.44
	CL(L/h/kg)	13,401.24 ± 12,509.88	7004.13 ± 4323.30	1314.13 ± 674.08	4760.87 ± 3179.04

t_1/2_, elimination half-life; C_max_, maximum plasma concentration; T_max_, time to maximum plasma concentration; AUC, area under the concentration–time curve; V_d_, volume of distribution; CL, clearance.

## Data Availability

The data presented in this work are available in the article.
